# Survival of Free and Encapsulated Human and Rat Islet Xenografts Transplanted into the Mouse Bone Marrow

**DOI:** 10.1371/journal.pone.0091268

**Published:** 2014-03-13

**Authors:** Raphael P. H. Meier, Jörg D. Seebach, Philippe Morel, Redouan Mahou, Sophie Borot, Laurianne Giovannoni, Geraldine Parnaud, Elisa Montanari, Domenico Bosco, Christine Wandrey, Thierry Berney, Leo H. Bühler, Yannick D. Muller

**Affiliations:** 1 Cell Isolation and Transplantation Center, Department of Surgery, Geneva University Hospitals and University of Geneva, Geneva, Switzerland; 2 Division of Clinical Immunology and Allergology, Department of Internal Medicine, University Hospital and Medical Faculty, Geneva, Switzerland; 3 Institut d’Ingénierie Biologique et Institut des Sciences et Ingénierie Chimiques, Ecole Polytechnique Fédérale de Lausanne, Lausanne, Switzerland; Children’s Hospital Boston, United States of America

## Abstract

Bone marrow was recently proposed as an alternative and potentially immune-privileged site for pancreatic islet transplantation. The aim of the present study was to assess the survival and rejection mechanisms of free and encapsulated xenogeneic islets transplanted into the medullary cavity of the femur, or under the kidney capsule of streptozotocin-induced diabetic C57BL/6 mice. The median survival of free rat islets transplanted into the bone marrow or under the kidney capsule was 9 and 14 days, respectively, whereas that of free human islets was shorter, 7 days (bone marrow) and 10 days (kidney capsule). Infiltrating CD8^+^ T cells and redistributed CD4^+^ T cells, and macrophages were detected around the transplanted islets in bone sections. Recipient mouse splenocytes proliferated in response to donor rat stimulator cells. One month after transplantation under both kidney capsule or into bone marrow, encapsulated rat islets had induced a similar degree of fibrotic reaction and still contained insulin positive cells. In conclusion, we successfully established a small animal model for xenogeneic islet transplantation into the bone marrow. The rejection of xenogeneic islets was associated with local and systemic T cell responses and macrophage recruitment. Although there was no evidence for immune-privilege, the bone marrow may represent a feasible site for encapsulated xenogeneic islet transplantation.

## Introduction

The incidence of type 1 diabetes is constantly rising in children and adolescents since the mid-1950’s [1], [2]. Allotransplantation of pancreatic islets is currently an option for the treatment of diabetic type-I patients suffering from repetitive and severe hypoglycemic episodes. However, the possibility of receiving an islet transplant is limited mainly due the shortage of organ and the need for life-long immunosuppression. The utilization of islets from other species (xenograft) and administration in encapsulated form represent attractive strategies to overcome both problems.

Clinical trials of allogeneic human or xenogeneic pig encapsulated islets have been reported in only a few cases [3], [4]. After intra-peritoneal transplantation all patients showed a modest reduction in insulin requirement and a significant diminution of hypoglycemic episodes without any detectable immune response against the islets. Nevertheless, sustained insulin-independence was not achieved after encapsulated allogeneic islet transplantation. In addition, blood porcine C peptide levels remained low or undetectable suggesting graft failure. Certain investigators are of the opinion that this poor outcome is related to the high sensitivity of pancreatic islets to hypoxia [5]. Indeed, islets are highly vascularized structures depending on an arterial oxygen supply of 40 mmHg [6]. The peritoneum, although it allows the implantation of a large volume of islets, suffers from low oxygen pressure and limited vascularization [7], [8].

Recently the bone marrow (BM) has been proposed as an alternative site for autologous and allogeneic islet transplantation because it is considered to be well-vascularized and easily accessible [9], [10]. Syngeneic islets reverse diabetes without compromising the hematopoietic activity [11]. Recently, The group of Piemonti has reported the first unequivocal example of successful engraftment of autologous islets in human BM for up to 944 days [9]. While direct differentiation of hematopoietic stem cells into islet cells is highly unlikely, a wide array of experimental evidences indicates that cells of BM origin are capable of facilitating the survival, reorganization and revascularization of the islets [12]–[15]. Finally, it has been suggested that BM is an immune privileged site. Histological findings in the rat tibia showed intact islet allografts three weeks after transplantation without any immunosuppression [16]. Thus, the BM could represent a potential alternative transplantation site for encapsulated islet xenograft.

The aim of this study was to establish an animal model for xenogeneic islet transplantation into BM. We assessed the survival of xenogeneic rat and human pancreatic islets transplanted into the BM of mice and characterized the ensuing immune responses. In addition we investigated the feasibility of transplanting encapsulated Sprague Dawley rat islets to the femur of C57BL/6 mice. It should be noted that *in vivo* functional assays with encapsulated islets under the kidney capsule (KC) or into the BM were not feasible due to space limitations.

## Materials and Methods

### Animals

Pancreatic islets were harvested from adult Sprague-Dawley rats with a weight of 250–300 grams and transplanted into C57BL/6 mice between 6–10 weeks of age (Centre de Recherche et d’Elevage, Janvier, France). Animals were maintained in conventional housing facilities and all experiments were performed in compliance with the bylaws of Geneva veterinary authorities and were approved by the ethical committee of the Geneva University Medical School (Protocol Nr. 1014/3767/2).

### Human Pancreatic Islets

Human islets were isolated according to the Ricordi protocol with local adaptation [17]. Human islets used in this study had a minimum purity of 80% as assessed with Metamoph software (MetaMorph, Universal Imaging, West Chester, PA) as previously described [18]. The use of human islet preparations for experimental research was approved by the Institutional Review Board for clinical research of the Departments of Neurology, Dermatology, Anesthesiology and Surgery of the University Hospital of Geneva (CER Nr. 05–028). Our ethical institution waived the need for consent from the donor. Islets were used for experimental research only when not suitable for clinical purposes and with the intention to be destroyed. In such cases, obtaining informed consent is not mandatory in Switzerland. Tissues samples were not procured from a tissue bank.

### Rat Pancreatic Islets

Rat pancreases were perfused with collagenase type XI, 1 mg/ml (Sigma, Buchs, Switzerland), removed surgically, and enzymatically digested in vitro at 37°C for 10 minutes as previously described [19]. Islets were then purified by Ficoll density gradient centrifugation and maintained on ice prior to transplantation.

### Islet Transplantation

Diabetes was induced in the recipient mice by a single intraperitoneal (i.p.) injection of streptozotocin (Sigma, Buchs, Switzerland), 220 mg/kg. Only diabetic mice with blood sugar levels of >20 mmol/L were used for the transplantation of 1000 rat and 3000 human islets equivalent (IEQ), either into the BM or under the kidney capsule (KC) of diabetic mice. Higher numbers of human islets are required to reverse hyperglycemia due to murine resistance to human insulin [20]–[21]. Islets were compacted in a pellet and rapidly transplanted into the BM or under the KC as prolonged compaction of the islets can affect their survival [22].

**Figure 1:**
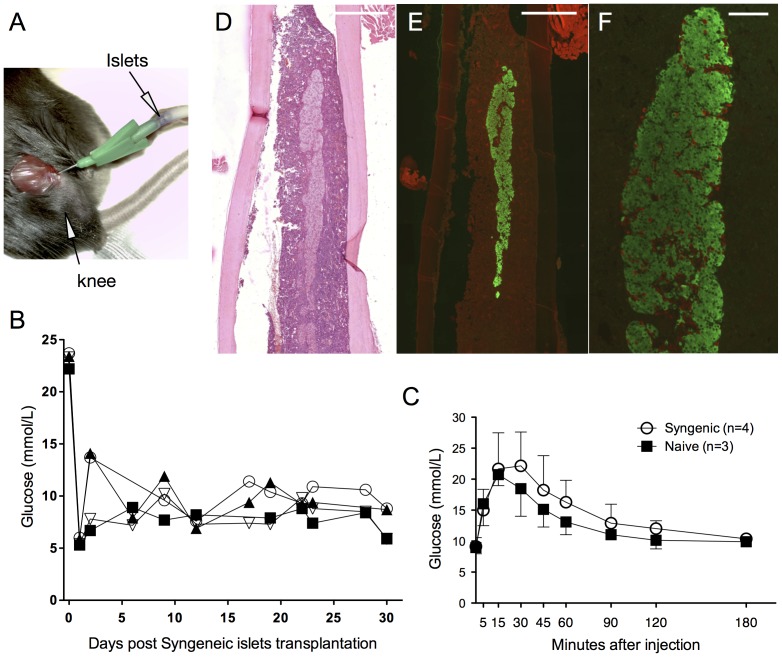
Transplantation of syngeneic islets into the bone marrow. A 22-gauge needle was used for injection of the syngeneic islets into the bone marrow of C57BL/6 through the distal part of the femur (A). Transplanted mice remained normoglycemic over 30 days (B). Intraperitoneal glucose tolerance test was performed after 30 days in transplanted and naïve mice. Error bars represent the standard deviation. (C). Bones were harvested after 30 days and stained for haematoxylin and eosin (D), insulin (E) and glucagon/insulin (F). Glucagon positive cells appear in red whereas insulin positive cells appear in green. Scale bar 500 µm (D,E), 100 µm (F).

In accordance with the 3R principles of animal experimentation (reduce, refine, replace), only half of the rat islets were transplanted in experiments using non-diabetic mice. Islets were inserted in a 22-gauge butterfly as previously described [19]. For transplantation into the BM a medial incision was made to the anterior surface of the knee of the mice. A 29-gauge needle followed by a 22 gauge was inserted into the distal part of the femur to prepare the pathway for the islets. The islets were slowly injected using a 22-gauge needle (Figure 1A). After careful retraction of the injection needle the skin was closed with 5.0 sutures. Islet graft function was assessed by regular blood sugar determination (Precision Q.I.D). Blood sugar levels of <13 mmol/l were considered as successful islet transplantation, whereas levels of >20 mmol defined graft failure.

### Islet Encapsulation

Encapsulation in Ca-alginate was performed as previously described [23]. Briefly, a stock solution of ultrapure sodium alginate (Na-alg, Pronova UP LVM; batch No FP-506-01, FMC BioPolymer, Norway) was prepared with a final concentration of 1.5% w/v in Dulbecco’s Modified Eagle Medium (DMEM, Cell Culture Technologies LLC, Gravessano, Switzerland). The islets were suspended in the 1.5% Na-alg stock solution (10′000 IEQ/ml), homogenized, and finally extruded into the gelation bath (CaCl_2_, 110 mM) using a coaxial airflow droplet generator (Buchi, Basel, Switzerland). Beads were gelled at 37°C for 15 min, collected by filtration, washed twice with DMEM, and cultured at 37°C. Encapsulated islets were transplanted using an 18 gauge needle. The average bead size was in the range of 450–500 µm.

### Histological Analyses

#### Paraffin

For morphologic evaluation kidney samples were preserved in formalin 10%, fixed in 4% paraformaldehyde, decalcified, embedded in paraffin and sectioned. Paraffin sections (5 um) were used for haematoxylin and eosin (H&E) staining. Paraffin sections were incubated using guinea pig anti-insulin antibody (1∶100 dilution, Invitrogen, Basel, Switzerland) and rabbit anti-glucagon (dilution 1∶200, Dako, Denmark), and subsequently with goat anti-guinea pig IgG Alexa 488-conjugated (1∶1000 dilution, Invitrogen) and anti-rabbit IgG Alexa 566 (Dilution 1∶1000, Life Technologies, Carlsbad, CA). Alternatively, paraffin sections were stained with guinea-pig anti- insulin antibody, subsequently anti-guinea-pig IgG Alexa 488, and thereafter with 0.09% Evans blue at the termination of the procedure.

#### Cryostat

Kidney and BM samples were harvested three days following islet transplantation, stored at –80°C and used for immunofluorescence staining. Briefly, frozen sections were incubated for two hours with rat anti-mouse CD4 (550278, 1∶50 dilution, Becton Dickinson), rat anti-mouse CD8 (MCA 609G, 1∶100 dilution, Serotec), or F4/80 antibodies (MCA 497, 1∶50 dilution, Serotec) to detect macrophages. Secondary staining was performed with anti-rat IgG Alexa 555-conjugated (1∶1000 dilution, Invitrogen). Thereafter, the slides were stained for insulin as described for the paraffin section (1∶1000 dilution, Invitrogen). The sections were then mounted and viewed under a fluorescent microscope (Zeiss).

### Flow Cytometry

Seven days post-transplantation, femurs bearing islet grafts, contralateral femurs, and spleens were processed for flow cytometry analysis. Naive mice were used as negative controls. Bones were flushed with PBS and spleens were smashed with the back of the piston of a 1 ml syringe. Red blood cells were lysed before counting (Pharm Lyse BD Bioscience San Jose CA). Due to the techniques used for cutting and flushing the bones, the absolute number of cells harvested was variable between samples (Figure S1). Samples of 200’000 BM cells or splenocytes were stained with anti-CD4 (APC Cy7, RM4–5 eBioscience San Diego, CA), anti-CD8 (PerCP 53-6.7, eBioscience), and F4/80 antibodies (APC, CI:A3-1, Serotec). Irrelevant isotype control antibodies included rat IgG2b APC-Cy7, rat IgG2a PerCP, and rat IgG2b APC. Data were collected on a FACScanto (Becton Dickinson, Franlin Lakes, NJ). All data were analyzed using Flowjo software (Tree Star v. 8.7.3, Ashland, OR).

### Mixed Lymphocyte Reaction

One-way mixed lymphocyte reactions were performed seven days post-transplantation between donor Sprague Dawley rat T-cell-depleted splenocytes and recipient C57BL/6 splenocytes. Briefly, rat donor splenocytes were harvested on the same day as the islets. After red blood cell lysis, the cells were stained with mouse anti-rat CD3-PE antibody (OX-38, BD bioscience) followed by negative selection using LD columns (Miltenyi Biotec, Bergisch Gladbach, Germany). This procedure resulted in an over 95% pure CD3–depleted cell population which was frozen until seven days post islet transplantation. A total of 2.5×10^5^ splenocytes obtained from transplanted or naive control mice were co-cultured with 4×10^5^ irradiated (3500 rad) T cell-depleted rat donor splenocytes for 5 days. Cells were then pulsed with 1 µCi^3^[H] of thymidine for 18 hours and harvested.

### MetaMorph Quantification

The total surface of positive collagen staining on bone and kidney sections was determined using the morphometric quantification software (Universal Imaging). Thus, the blue area (collagen I to VI) was normalized to the red area (cells) on Masson’s trichrome staining and the number of capsules. Bone surfaces, which constitutively stain blue, were excluded. Alternatively, the number of CD4, CD8 and F4/80-positive cells was counted at 200× magnification on images of bone sections (8–11 fields per group out of three animals) using offline MetaMorph imaging software for microscopy. The number of positive cells was normalized with the total number of cells per 200× magnification fields (Hoechst positive cells counted using MetaMorph software) and expressed as a percentage.

### Insulin Secretion test

This test was performed using encapsulated rat islets after 30 days of culture. Insulin release in response to acute glucose stimulation was determined by incubating the islets for 1 hour in RPMI 10% FCS medium containing low glucose (2.8 mM) for basal secretion, followed by an additional hour of incubation in high glucose (16.8 mM) medium or 16.8 mM glucose medium supplemented with 5 mM of theophylline. Supernatants were collected, frozen and insulin concentrations were determined using rat insulin ELISA Kit according to the manufacturer’s instructions (Mercodia, Uppsala, Sweden). Results were normalized to the total insulin content of the islets as measured by the same ELISA kit and following ice-cold acid-ethanol extraction. Due to inter-experimental variability, and depending upon the time of culture and the encapsulation, insulin secretion was expressed relative to the basal levels (stimulation index).

### Intraperitoneal Glucose Tolerance Test (IPGTT)

Animals were maintained on a standard diet overnight, and then injected intraperitoneally with a 2 g/kg glucose solution re-suspended in PBS. Blood samples were collected after 0, 5, 15, 30, 60, 90, 120 and 180 minutes.

### Statistical Analysis

Prism software was used for statistical analysis (GraphPad Software, San Diego California, USA). Survival curves were calculated by the Kaplan and Meier method and analyzed using the Cox-Mantel test. Flow cytometry results were analyzed using the Krukal-Wallis and Dunn’s multiple comparison tests. The Mann-Whitney U test was used for mixed lymphocyte reaction and MetaMorph quantification. A p-value inferior to 0.05 was considered statistically significant.

## Results

### Injection of Islets into the Bone Marrow

Syngeneic islets were injected into the femur of diabetic mice through the knee (Figure 1A). The transplanted mice remained normoglycemic indefinitely (Figure 1B). Sham transplantation into the bone marrow did not alter the weight gain of the mice as compared to naive mice (Figure S2). Intraperitoneal glucose tolerance testing was performed on day-30 post-transplantation and showed a similar glucose tolerance profile between transplanted and naive control mice (Figure 1C). Thirty days after transplantation bone was harvested and stained for H&E (1D), insulin (1E) and insulin/glucagon (1F). Of note, islets must be slowly injected into the BM since rapid injection may provoke pulmonary embolism and consequently the death of the animal (Figure S3). Removal of the graft-bearing femur resulted in animal death rapidly after the procedure, before reversion to hyperglycemia (n = 4, data not shown).

### Survival of Concordant Rat and Discordant Human islets Xenograft

**Figure 2:**
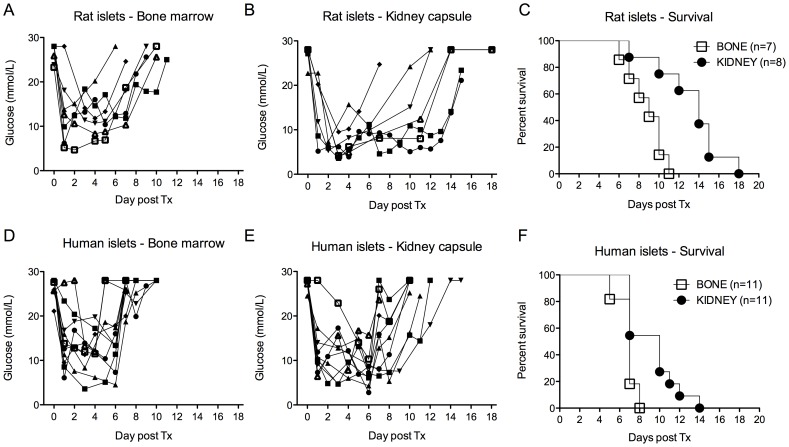
Free xenogeneic islet survival. Streptozotocin-induced diabetic mice were transplanted with rat (A-B-C) or human (D-E-F) pancreatic islets either in the bone marrow (BM) or under the kidney capsule (KC). The glycemic profile was followed over time in all groups. The median survival of rat islets was 9 days when transplanted into the BM (n = 7) and 14 days when transplanted under the KC (n = 8) (A-B-C). The median survival of discordant human islets was shorter in both sites with 7 and 10 days respectively in the BM (n = 11) and under the KC (n = 11) (D-E-F).

We next investigated the survival of concordant rat and discordant human islets transplanted into the BM and KC of streptozotocin-induced C57BL/6 diabetic mice. One thousand rat islets equivalent (IEQ) and 3000 human IEQ were used for prompt reversal of diabetes after transplantation into the BM (Figure 2A–F). The median survival of free rat islets was 9 days when transplanted into the BM (n = 7) and 14 days when transplanted under the KC (n = 8) (2A–C). The median survival of discordant human islets was shorter at both sites, 7 and 10 days respectively in the BM (n = 11) and under the KC (n = 11) (2D–F). Thus, there was no evidence for an immune-privileged environment in the BM for neither concordant nor discordant islet xenograft.

### Characterization of Islet Graft Rejection in the Bone Marrow

**Figure 3:**
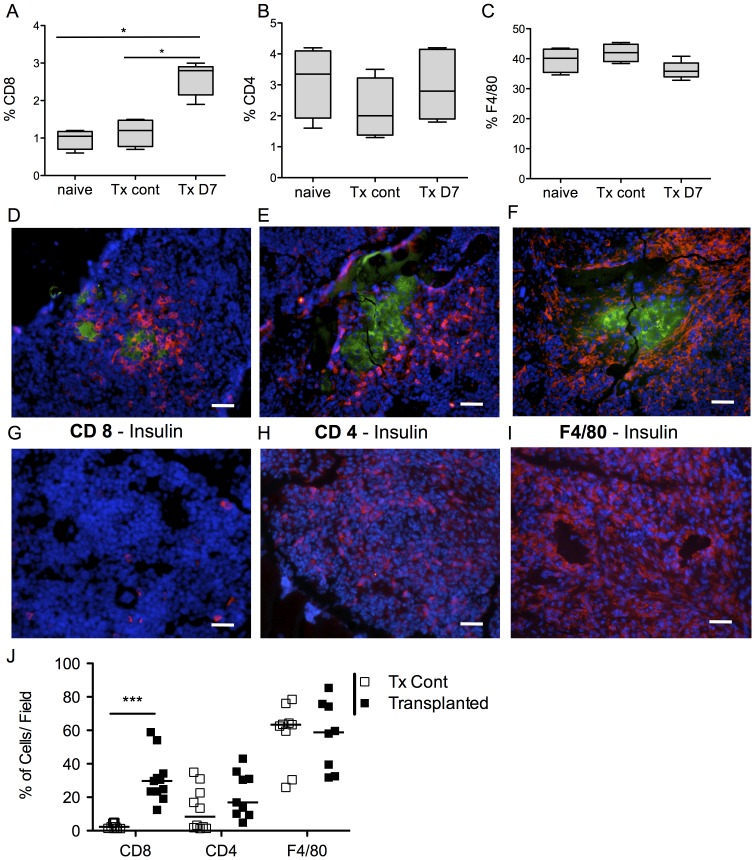
Characterization of the rejection in the bone marrow after rat islets transplantation. (A–C) Seven days post-transplantation, animals were sacrificed for the characterization of rejection. The graft-bearing femur (n = 5), the opposite control femur (n = 4) and the femur of non-transplanted animals (n = 4) were harvested, flushed and the cells analyzed by flow cytometry. The percentage of CD8^+^ (A) cells in transplanted femurs was significantly increased whereas percentages of CD4^+^ (B) and F4/80^+^ (C) cells remained unchanged compared to the non-transplanted contralateral control femurs. Box-and-whisker diagram are shown. (D–F) Alternatively, the femurs of graft bearing-femurs (D–F) and the contralateral femurs (G–I) were frozen in liquid nitrogen and double stained for insulin (green) and CD8 (D/G), CD4 (E/H) or F4/80 (F/I) (red) three days post transplantation. Scale bar 50 µm. (J) CD4^+^, CD8^+^ and F4/80^+^ cells were quantified using the MetaMorph software. The levels of CD4^+^ and F4/80^+^ cells were similar between both groups; CD8^+^ cells were significantly more abundant in femurs containing the islet graft. Abbreviation. Tx Cont: contralateral femur. TX D7: graft bearing femur.

Due to the immunosuppressive effects of the streptozotocin [24] we further characterized the rejection of free rat islets in non-diabetic mice. By flow cytometry we first assessed the percentages of CD4^+^, CD8^+^, and F4/80^+^ cells in the BM. The graft-bearing femurs and the contralateral femur of transplanted mice were harvested seven days post-transplantation and flushed. Femurs of non-transplanted naive mice were also used as controls. Absolute numbers of cells in the femurs of the three groups were similar (Figure S1). Within the total cell population we found a significant increase of the percentage of CD8^+^ cells in transplanted femurs, whereas percentages of CD4^+^ and F4/80^+^ cells remained unchanged (Figure 3A–C). Three days post-transplantation the graft bearing-femurs (3D–3F) and the contralateral femur (3G–I) were frozen in liquid nitrogen and double stained for insulin and CD8 (3D/G), CD4 (3E/H) or F4/80^+^ (3F/I). Densities of CD4^+^, CD8^+^ and F4/80^+^ cells were markedly increased around islets (Figure 3D–F). Quantitative data of the immunochemistry staining revealed that the amount of CD4^+^ and F4/80^+^ cells were similar between both groups and that CD8^+^ cells were more abundant in femurs containing the islet graft compared to controls (Figure 3J). Altogether, these results suggest that islet transplantation induced the proliferation of CD8^+^ cells in the BM, and rejection was mediated at least partly through the recruitment of CD4^+^, CD8^+^ and macrophages.

### Antigen Recognition in the Spleen after Xenogeneic Islet Transplantation

**Figure 4:**
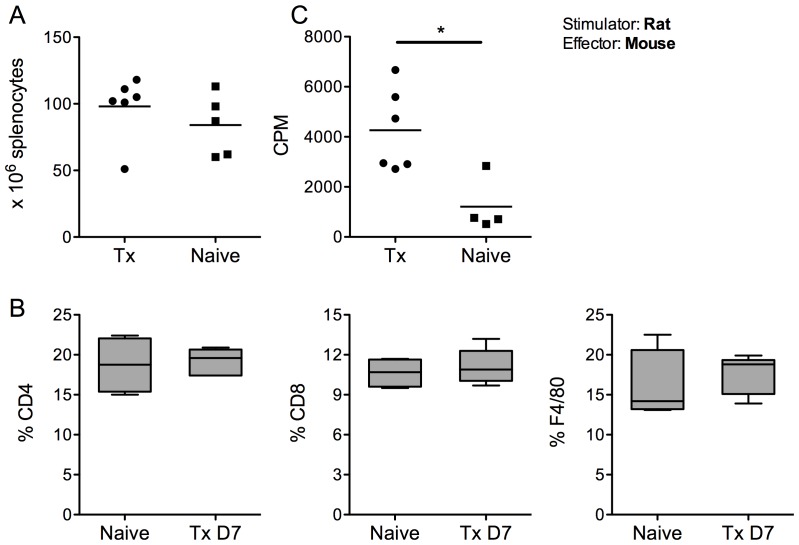
Characterization of the splenocytes after rat islet transplantation into the bone marrow. The splenocytes of transplanted and naive animals were harvested seven days after transplantation. The absolute number of splenocytes was not significantly different compared to naive mice; data are shown for individual animals with the horizontal line representing the mean value (A). The percentages of CD4^+^, CD8^+^ and F4/80^+^ cells were not significantly different either. Box-and-whisker diagram are shown (B). However, splenocytes of transplanted mice showed a significantly increased level of proliferation compared to naive mice when stimulated by donor (T cell-depleted) rat irradiated splenocytes. Results are expressed in counts per minute and show the mean value of 4 naive and 6 transplanted animals (C). Abbreviation. Tx: transplanted. CPM: Count per minutes.

Because BM itself is a lymphoid tissue the immune response induced by the presence of xenogeneic islets could remain localized in the femur. To assess a potential systemic immune response we harvested the spleen of non-diabetic mice for phenotypic and functional assays seven days after rat islet transplantation. The absolute number of harvested cells was not significantly different compared to naive mice (Figure 4A). The percentages of CD4^+^, CD8^+^ and F4/80^+^ cells were also not significantly different (Figure 4B). However, splenocytes of transplanted mice compared to naive mice showed a significantly increased proliferation when stimulated by donor (T cells-depleted) rat splenocytes (Figure 4C), suggesting that antigenic recognition of the xenogeneic islets occurred in the spleen.

### Encapsulated Rat Islets Survived More than One Month *in vitro* and in Bone Marrow

**Figure 5:**
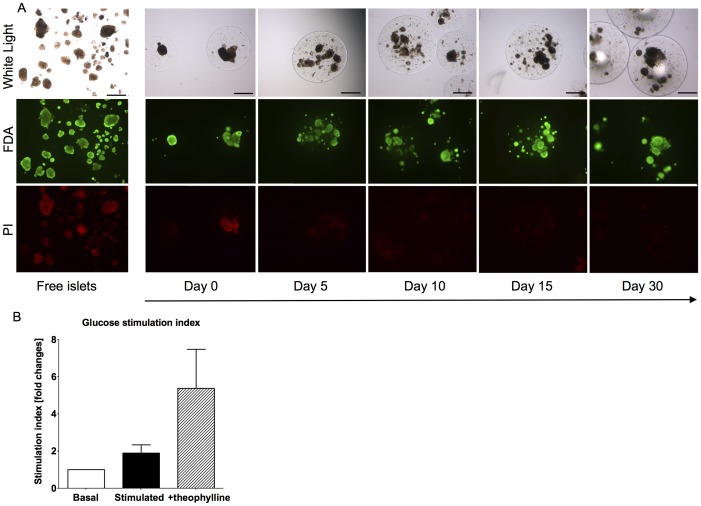
Survival and function of encapsulated rat islets *in vitro*. Viability was assessed over 30 days in(green) and propidium iodide (red). Encapsulated islets remained viable over 30 days of culture. Scale bar 200 µm (A). The function of the encapsulated islets was tested in vitro 30 days post encapsulation by glucose stimulated insulin secretion test. Islets were incubated in medium with 2.8 mM glucose, 16.8 mM glucose and 16.8 mM glucose supplemented with theophylline. The stimulation index was respectively 2 (glucose-stimulated) and 5 (theophylline-stimulated). The mean and SEM are shown of one representative experiment out of three (B).

**Figure 6:**
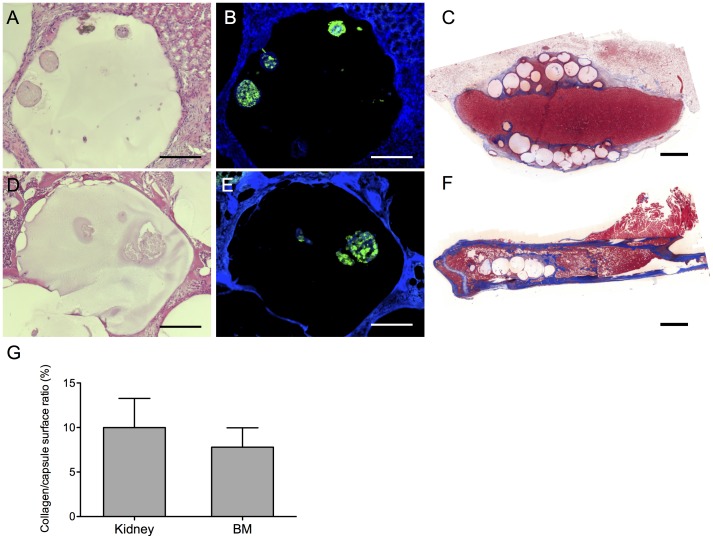
Encapsulated rat islets one month after transplantation. Encapsulated rat islets were transplanted under the kidney capsule (KC) (A–C) or into the bone marrow (BM) (D–F) One month after transplantation the femur and kidney were harvested. Haematoxylin and eosin staining showed intact capsules in the KC (A) and BM (D). Insulin staining confirmed the survival of the rat islets (B, E). The pericapsular fibrotic reaction was assessed by Masson’s coloration (C, F). The collagen/capsule surface ratio was quantified using MetaMorph software (G). Data presented are mean values ±SEM out of three (bone marrow) and four (kidney capsule) animals. Scale bar 100 µm (A,B,D,E), 1000 µm (C,F).

As a next step we assessed the feasibility of transplanting encapsulated rat islets into the BM. The viability of the islets was confirmed *in vitro* by fluorescein diacetate and propidium iodide up to 30 days after encapsulation (Figure 5A). At day 30 encapsulated islets still responded to glucose and theophylline stimuli (Figure 5B). We next transplanted encapsulated rat islets into the BM and under the KC of non-diabetic mice. After 30 days the femur and kidney were harvested, and H&E staining showed intact capsules at both sites (Figure 6A, 6D). Additionally, the islets in the capsules stained positive for insulin and DAPI suggesting that they survived *in vivo* (Figure 6B, 6E). *In vivo* functional assays could not be performed in the femur or under the KC because transplantation of sufficient numbers of encapsulated islets was not possible due to space limitations (data not shown). Finally, we quantified the fibrotic reaction against the capsules (Figure 6C, 6F) Similar levels of fibrosis were present under the KC and in the BM, after exclusion of the bone structures which constitutively contain collagen (Figure 6G). Of note, fibrosis does not develop after transplantation of syngeneic islets under the kidney capsule or into the BM (data not shown).

## Discussion

The overall goal of this study was to establish a small animal model to evaluate the BM as a potential transplantation site for free and encapsulated islet xenografts. The rational of transplanting islets into the BM is based on the accessibility of the site with the possibility for repeated infusions, and on the extravascular but well vascularized bone microenvironment [11]. The liver, the currently used site for islet transplantation, has several disadvantages including: (i) immediate blood-mediated inflammatory reaction (IBMIR) characterized by the activation of complement, platelets and coagulation as well as neutrophil recruitment, induces the loss of as many as 50–75% of islets during engraftment [25]; (ii) risk for hemorrhages associated with the infusion through the liver parenchyma into the portal vein; and (iii) increase of portal pressure during the procedure which limits both the number and mass of administered islets, and precludes the transplantation of encapsulated islets with a diameter up to 500 µm [26]. The emerging perspective of transplanting encapsulated xenogeneic, i.e. porcine islets, into humans prompted us therefore to analyze the BM as an alternative transplantation site.

Transplantation into the BM of 1000 rat IEQ and 3000 human IEQ per mice was required in order to reverse diabetes, which is 2–3 times higher than what is required for a xenogeneic islets transplant under the KC [19]. There are several reasons for this finding. First, the injection procedure through the spongy framework of the femur may be associated with loss due to substantial mechanical stress. Secondly, direct blood contact with the islets in the BM may provoke an IBMIR as occurs in the liver. Furthermore, IBMIR is even enhanced due to the xenogeneic tissue [27]. Finally, the release of insulin may be less efficient in the BM than under the KC. Nevertheless, as shown in a syngeneic mouse model, islets transplanted into the BM progressively normalized glycaemia with maximal function 3 to 4 weeks after transplantation, with a 2.4-fold higher probability to reach euglycaemia when compared to intraportal islet transplantation [11]. Of note, we cannot exclude that ischemia time and donor characteristics affected the function and viability of the human islets used in this study, and thus may have influenced the survival rates after transplantation. Altogether, our results confirmed that xenogeneic islets transplanted into the BM could normalize blood glycaemia in a xenogeneic model. However, further studies are still warranted in larger animal models to better define the advantages and limitations of the BM as a potential site for xenogeneic islets.

Our second aim was to analyze the rejection process of non-encapsulated xenogeneic islets transplanted into the BM. The median survival of free rat and human islets was shorter in the BM than under the KC, excluding any immune privileged environment in the BM [11]. In contrast to vascularized organs that are highly sensitive to antibody-mediated rejection through complement activation, xenogeneic islets are mainly rejected by cellular-dependent mechanisms [28]. In particular CD4^+^ T cells play a critical role in the initiation of a Th2-like immune response by the recruitment of macrophages [29]–[31]. CD8^+^ T cells are also capable of rejecting pancreas islet xenograft in the absence of CD4+ T cells [32] although it is not considered to be the *primum movens* for rejection of cellular grafts in xenotransplantation. Our study confirmed the presence of macrophages and CD4^+^ and CD8^+^ T cells infiltrating the islet xenograft, which suggests that the mechanisms responsible for rejection in the BM are similar to those that have been previously described. *In vivo* depletion studies would be required to differentiate the respective role of each cell subset knowing that the mechanisms of cell-mediated islet xenograft rejection are characterized by redundancy [28]. In addition, further analysis of the cellular surface markers and cytokines secretion profile would give more information of the role of each cell subsets during islet rejection into the BM.

Seven days after islet transplantation the spleens were harvested. There were no changes found in the frequencies of CD4^+^, CD8^+^ and macrophages, although splenocytes proliferated in response to donor antigen when compared to naive animals. This suggests that the islet xenograft elicited an immune response through CD4 priming in the spleen. Antigen recognition triggers xenograft immunity via direct pathways involving interspecies TCR/MHC interaction and indirect pathways through the presentation of xenogeneic peptides by recipient dendritic cells [33], [34]. In the absence of recipient antigen presenting cells (APC), the strength of the cell-mediated xenograft rejection becomes weaker suggesting that indirect antigen presentation is crucial for xenogeneic islet immunity [28]. Thus, our study confirmed that the immune response against xenogeneic islets was induced not only in the BM itself but also in secondary lymphoid organs such as the spleen. This could be mediated by passenger leukocytes migrating into the spleen or via free xenoantigens entering the peripheral circulation [35].

The last aim of this study was to assess the feasibility of transplanting encapsulated xenogeneic islets into the BM. Although the available space in the BM of mice was not sufficient to perform functional assays, insulin-positive islets could be retrieved one month after transplantation. The encapsulated islets were intact and did not induce a higher level of fibrosis as compared to the KC, despite the presence of an abundant cellular environment in the BM. Peri-capsular fibrotic overgrowth has been recognized as an important factor leading to failure of the encapsulated islets transplanted into the peritoneum [5], [36]. Fibrosis is induced by physical irregularities of the capsule [37], or by macrophages recruited via the chemokines secreted by the islets [36], [38]. To diminish the fibrotic reaction against the xenogeneic islets, the alginate beads could be chemically manipulated with polyethylene glycol reducing the pro-inflammatory properties associated with polycation-coated alginate microspheres [39], [40]. Altogether, the transplantation of encapsulated islets into the BM was feasible without inducing an extensive peri-capsular fibrosis. Nevertheless, further studies are necessary in a larger animal model with larger bones, such as the pig, in order to perform functional assays.

Except for the survival experiments that included rat and human islets, the analysis in the present study was based on the rejection of rat islets. Because only a few studies have compared the rejection of concordant (rat) and discordant (human) islets in the mouse model, it remains difficult to predict whether the present results for rat islets will be similar for human islets. First, the innate response composed by neutrophils, macrophages and natural killer cells plays an active role in discordant transplantation [41]. Second, the rejection depends on the ability of donor antigen presenting cells (APC) to efficiently present antigen to the helper and effector T cells. In discordant islet transplantation indirect presentation of antigens (by recipient APC) is a major pathway for islet rejection, whereas in concordant and allogeneic islets transplantation both recipient and donor APC participate in the rejection process [42]–[43]. In particular, the depletion of recipient macrophages (which include APC) prolongs the survival of human but not rat islets, suggesting a critical role for indirect antigen presentation in discordant transplantation [43]. Further studies are still warranted to compare rejection of concordant and discordant islets transplanted in the BM.

In conclusion, the present study demonstrated that the BM represents a potential alternative site for xenogeneic encapsulated islets. Caution has to be taken during islet injection due to the risk of pulmonary embolism. The mechanisms of xenograft rejection in the BM seemed to depend at least partially on macrophages and CD4^+^ and CD8^+^ T cells, with the caution that other cell types such as neutrophils or natural killer cells have not been analyzed and the antibody mediated rejection has not been evaluated. The physiological disproportion between the size of the murine femurs and the required volume of injected encapsulated islets did not allow us to study graft function in greater detail. Nonetheless, if these encouraging results can be reproduced in a large animal model they may open new perspectives for the transplantation of encapsulated xenogeneic islets into the BM in humans.

## Supporting Information

Figure S1 **Absolut numbers of cells harvested from the bone marrow.** The graft-bearing femurs and the contralateral femur of transplanted mice were harvested seven days post-transplantation and flushed. Femurs of non-transplanted naive mice were also used as controls. Due to technical issues, the absolute numbers of cells in the femurs were variable between samples. No significant differences were found between the groups.
(TIFF)

Figure S2 **Weight gain of mice after sham islet transplantation into bone marrow compared to naïve mice.** The weight of C57BL/6 mice following sham islet transplantation (white triangles) was monitored and compared to those of naïve mice (white squares). The transplantation procedure did not alter the weight of the mice.
(TIFF)

Figure S3 **Injection of islets into the bone marrow can provoke fatal pulmonary embolism.** Rapid injection of free islets into the bone marrow may be associated with fatal pulmonary embolisms. Air bubbles can be visualized through the inferior vena cava if injected rapidly into the femur (black arrow) (A). The lungs of the animals were harvested and stained with haematoxylin and eosin and showed solid aggregates in the pulmonary vessels (B). Haematoxylin and eosin staining, scale bar 100 µm.
(TIFF)
