# Supplementary figures and images for: Survival of Free and Encapsulated Human and Rat Islet Xenografts Transplanted into the Mouse Bone Marrow
Source: PLoS One. 2014 Mar 13;9(3):e91268. doi: 10.1371/journal.pone.0091268 (PMC3953382; doi:10.1371/journal.pone.0091268)

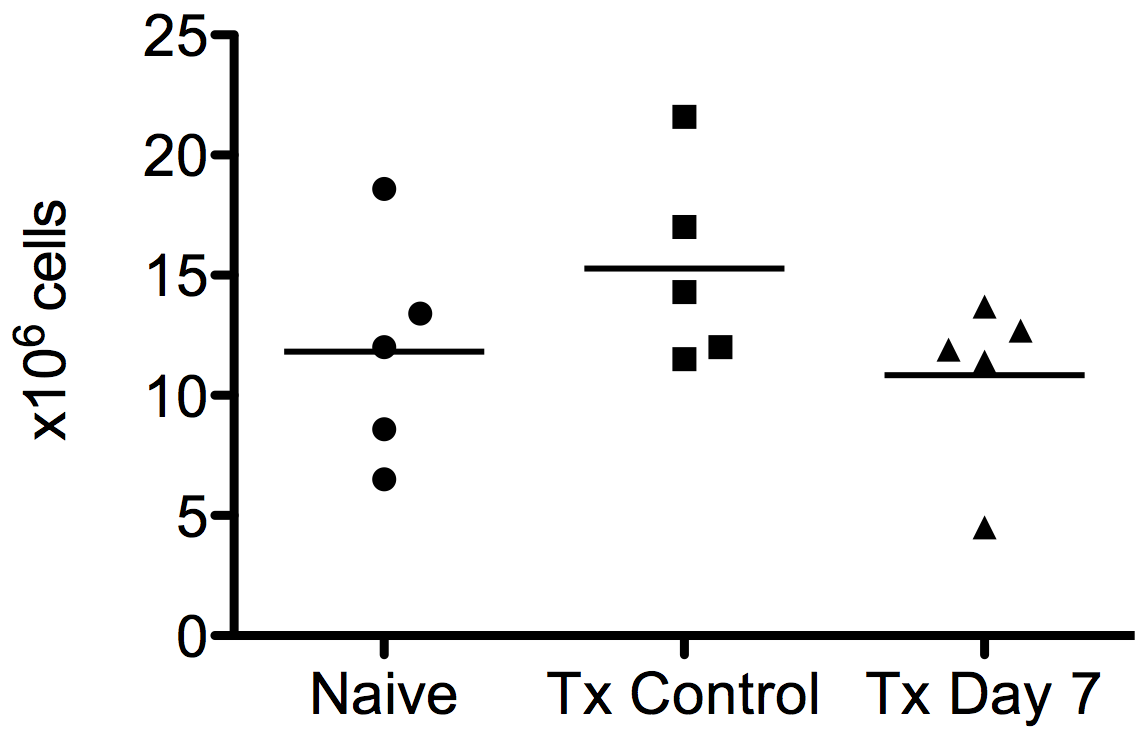

Supplement: Figure S1 — Absolut numbers of cells harvested from the bone marrow. The graft-bearing femurs and the contralateral femur of transplanted mice were harvested seven days post-transplantation and flushed. Femurs of non-transplanted naive mice were also used as controls. Due to technical issues, the absolute numbers of cells in the femurs were variable between samples. No significant differences were found between the groups. (TIFF) [file pone.0091268.s001.tif]

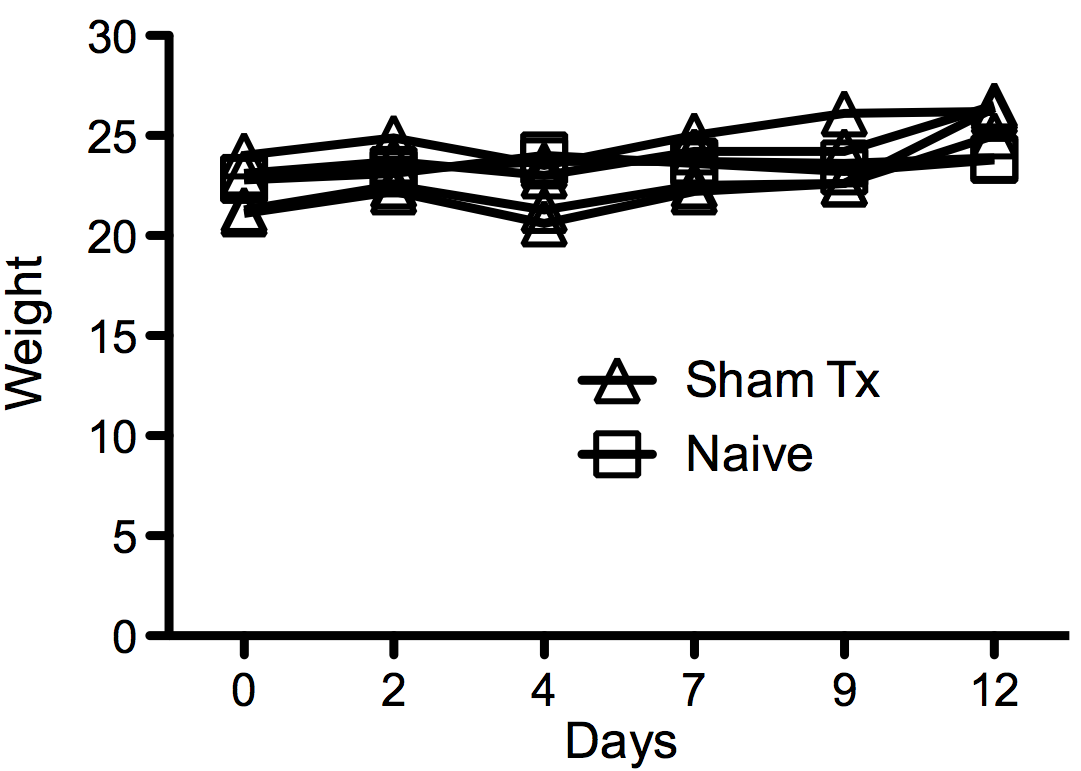

Supplement: Figure S2 — Weight gain of mice after sham islet transplantation into bone marrow compared to naïve mice. The weight of C57BL/6 mice following sham islet transplantation (white triangles) was monitored and compared to those of naïve mice (white squares). The transplantation procedure did not alter the weight of the mice. (TIFF) [file pone.0091268.s002.tif]

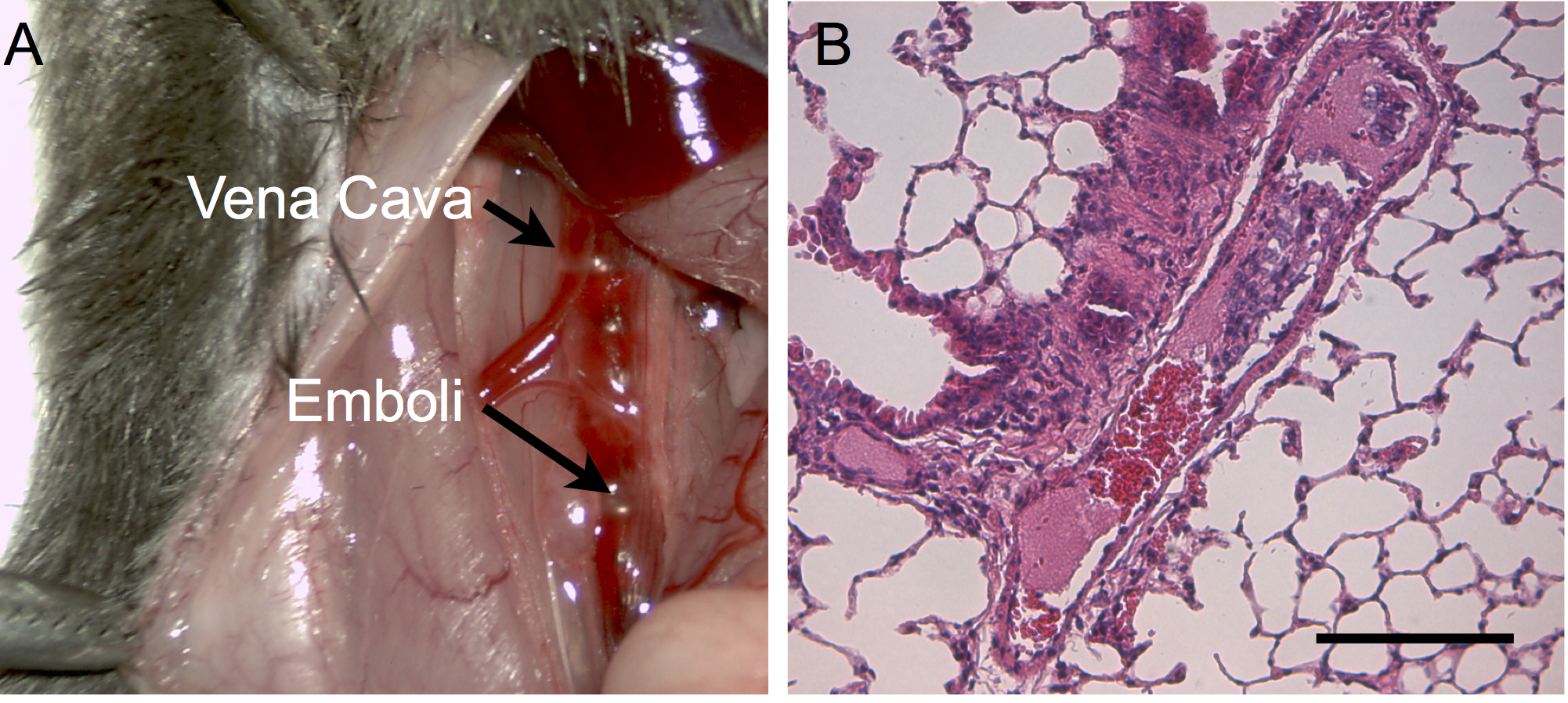

Supplement: Figure S3 — Injection of islets into the bone marrow can provoke fatal pulmonary embolism. Rapid injection of free islets into the bone marrow may be associated with fatal pulmonary embolisms. Air bubbles can be visualized through the inferior vena cava if injected rapidly into the femur (black arrow) (A). The lungs of the animals were harvested and stained with haematoxylin and eosin and showed solid aggregates in the pulmonary vessels (B). Haematoxylin and eosin staining, scale bar 100 µm. (TIFF) [file pone.0091268.s003.tif]
